# Case Report: Laryngospasm as Initial Manifestation of Amyotrophic Lateral Sclerosis in a Long-Survival Patient With Heterozygous p.D90A – *SOD1* Mutation

**DOI:** 10.3389/fneur.2021.708885

**Published:** 2021-09-30

**Authors:** Giuliana Capece, Mauro Ceroni, Enrico Alfonsi, Ilaria Palmieri, Cristina Cereda, Luca Diamanti

**Affiliations:** ^1^Department of Brain and Behavioral Sciences, University of Pavia, Pavia, Italy; ^2^General Neurology Unit, IRCCS Mondino Foundation, Pavia, Italy; ^3^Clinical Neurophysiology Unit, IRCCS Mondino Foundation, Pavia, Italy; ^4^Genomic and Post-genomic Centre, IRCCS Mondino Foundation, Pavia, Italy; ^5^Department of Molecular Medicine, University of Pavia, Pavia, Italy; ^6^Neuro-Oncology Unit, IRCCS Mondino Foundation, Pavia, Italy

**Keywords:** Amyotrophic Lateral Sclerosis, *SOD1*, p.D90A, laryngospasm, case report

## Abstract

Amyotrophic Lateral Sclerosis (ALS) is a fatal neurodegenerative disease affecting motor neurons. Although its etiology is still unknown, many genes have been found to be implicated in ALS pathogenesis. The Cu/Zn superoxide dismutase (*SOD1*) gene was the first to be identified. Currently, more than 230 mutations in the *SOD1* gene have been reported. p.D90A (p. Asp90Ala) is the most common *SOD1* mutation worldwide. It shows both autosomal and recessive inheritance in different populations. To date, five Italian patients with the heterozygous p.D90A mutation have been reported. None of them complained of laryngological symptoms as the initial manifestation of ALS, although they had atypical clinical features. We describe a long-survival patient carrying heterozygous p.D90A mutation who presented with severe laryngospasm due to bilateral vocal cord paralysis. We suggest that genetic analysis may help to diagnose ALS with insidious onset like hoarseness, laryngospasm, and other type of voice disturbances.

## Introduction

Amyotrophic Lateral Sclerosis (ALS) is a neurodegenerative disorder that affects the upper and lower motor neurons in the spinal cord, brainstem, and motor cortex (1). Its etiology is still unknown, but it is now accepted to be based on a complex interplay between environmental factors and genetic background (1). Most cases of ALS are sporadic (SALS), while about 10% of cases are familial (FALS), even though SALS and FALS are clinically identical and have both a genetic basis (1). In 1993, Rosen et al. discovered the first ALS-associated gene, *SOD1*, that is located on chromosome 21q22.11 and encodes a Cu/Zn-binding superoxide dismutase (2–4). To date, more than 230 mutations have been reported to be ALS-associated, but it is still controversial whatever all of them are disease-causative (3, 4).

Currently, p.D90A (p.Asp90Ala) is the most common *SOD1* mutation (3, 4) and is inherited as both a recessive and dominant trait in different populations (5). In northern Scandinavia, p.D90A heterozygous carriers are unaffected, while they developed ALS in Belgium (5).

In Italy, five patients carrying the heterozygous p.D90A mutation have been reported (6–9).

We herein report the heterozygous p.D90A mutation in a long-survival patient with ALS and laryngospasm as the initial manifestation.

## Case Presentation

A 58-year-old male was admitted to our department in May 2016 because of a 12-year history of progressive muscle weakness in the upper limbs. Family history for neurodegenerative diseases was negative. He was diagnosed with Gilbert syndrome and mild tricuspid valve regurgitation and he suffered from tachyarrhythmia and dyspepsia due to esophagitis. He presented with hypophonia due to a bilateral vocal fold paralysis occurred in 1999. Electromyography (EMG) showed a bilateral axonal neuropathy involving the superior and recurrent laryngeal nerves, especially on the left. As laryngeal spasms caused episodes of respiratory failure, he underwent a left laser posterior cordotomy. He began to suffer from low back pain after an accidental fall due to a disc herniation at L5-S1. Furthermore, mild postural tremor affecting the hands appeared and neurological examination showed muscle weakness in the proximal segments of the upper limbs. He was initially diagnosed with spinobulbar atrophy. Genetic tests for Kennedy's disease were negative. Furthermore, Brown-Vialetto-Van Laere syndrome was ruled out ex juvantibus by administering high-dose riboflavin and then by molecular analysis.

At first neurological examination, he had bilateral muscle weakness and hypotrophy of the proximal segments of the upper limbs, especially of the deltoids (Medical Research Council, MRC 3+ on the right side, 4− on the left side), of the both biceps brachii (MRC 3+) and triceps brachii (3−). Muscle strength was preserved in the distal segments of the arms. Triceps reflex was absent, while the other tendon reflexes of the upper limbs were normal. Lower limbs show no pathological signs except bilaterally decreased ankle jerk reflexes. Flexor plantar responses were assessed bilaterally. No fasciculations were detected. Cranial nerves were normal and there are no signs of sensory impairment. Magnetic Resonance Imaging (MRI) of the chest and cervical spine showed bilateral changes in the muscles of the rotator cuff, especially on the right, depending on a chronic neurogenic damage, and degeneration of the inferior roots of the brachial plexus. EMG and electroneuronography (ENoG) revealed diffuse motor axonopathy in spinal and bulbar regions with fasciculation potentials and denervation activity at rest, especially in the right deltoid, in the left thyroarytenoid muscle and in the thoracic paraspinal muscles on both sides. Sensory evoked potentials (SEPs) were normal. Routine laboratory investigations showed high levels of serum creatine kinase (359 UI/l; n.v.: 39–308). A diagnosis of motor neuron disease with predominant lower motor neuron phenotype was made.

**Figure 1:**
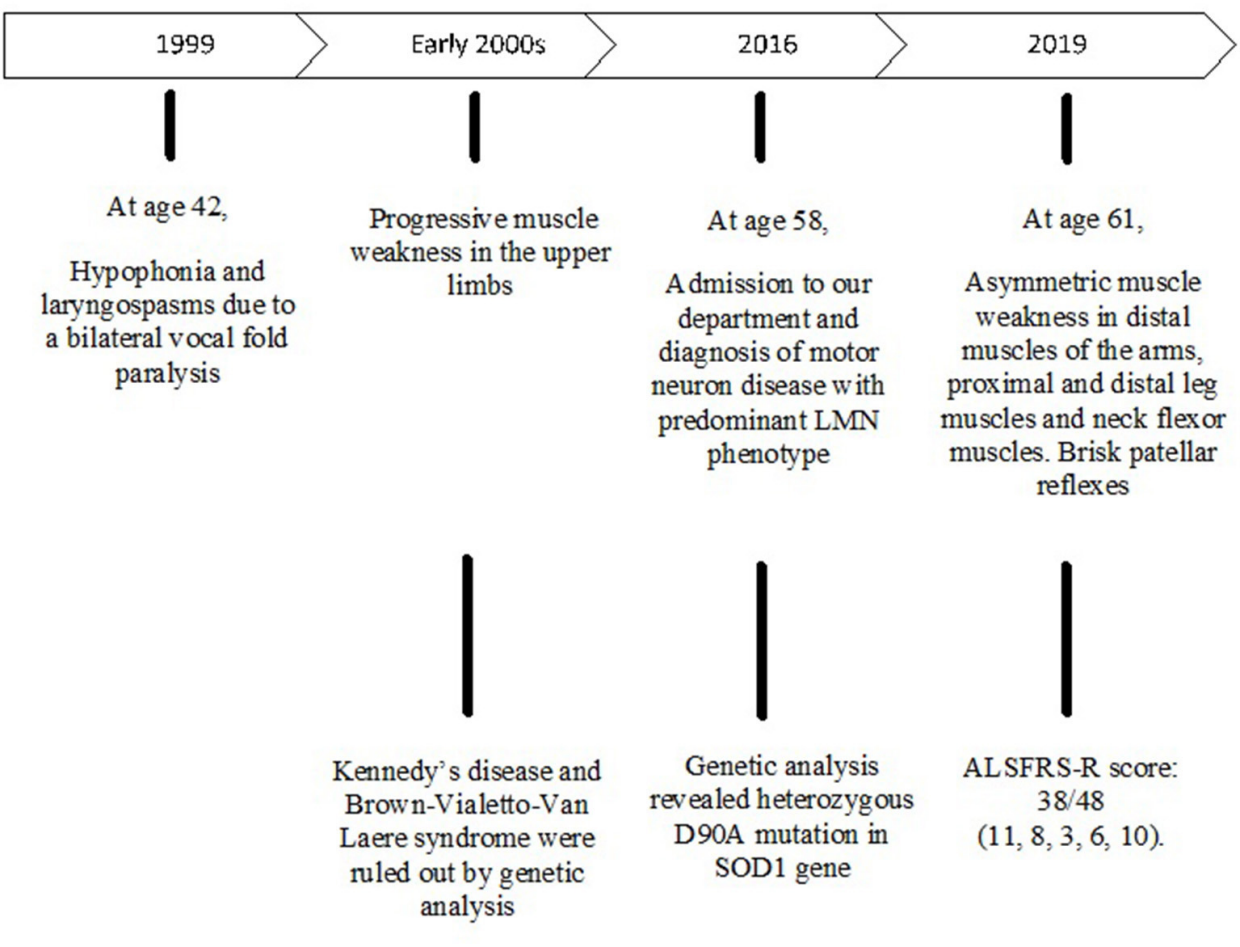
Sanger sequencing electropherogram; “*” shows the variant c.272A>C in the exon 4 of *SOD1* in the patient.

The patient's disease progression was monitored at 3-month intervals by testing muscle strength and performing ALSFRS-R (ALS-Functional Rating Scale Revised). Disease course was slowly progressive and he was clinically stable (Figure 1).

After a 3-year follow up, clinical examination showed weakness of neck flexor muscles (MRC 4), of proximal segments of the upper limbs (MRC 3–4), of the opponens pollicis muscle bilaterally (MRC 4), of the left iliopsoas, of the left peroneal muscles and of the left plantar flexors muscles (MRC 4, 5). Right plantar response was indifferent, while patellar reflexes spread to adductor muscles of the thigh. He complained of dysphagia and weight loss. According to the revised El Escorial criteria, a diagnosis of probable ALS-laboratory supported was made. The ALSFRS-R score was 38/48 (4, 7, 9–11).

## Genetic Analysis

**Figure 2:**
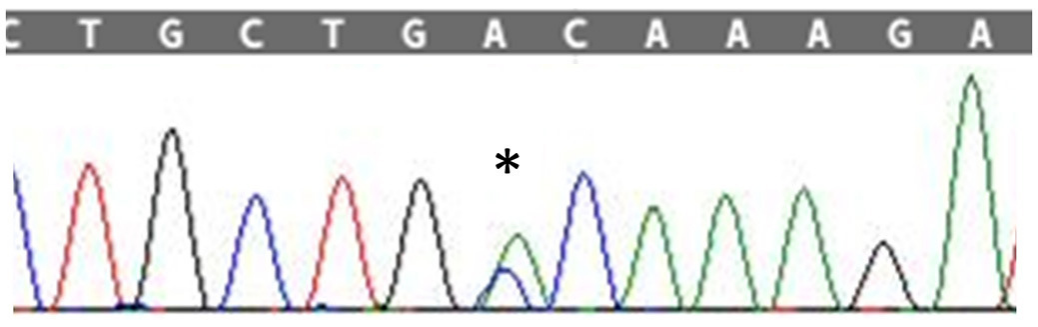
Timeline with relevant data from the episode of care.

After obtaining a written informed consent, Next Generation Sequencing (NGS) analysis was performed, using a customized panel of 174 genes related to neurodegenerative diseases as described in the Supplementary Material. We identified the heterozygous variant g.12669A>C, c.272A>C in the exon 4 of the *SOD1* gene resulting in the amino acid change p.Asp90Ala (Figure 2). The c.272A>C mutation was then confirmed by Sanger sequencing.

## Discussion

In this study we described an ALS patient presenting with laryngospasm as disease onset and a predominant lower motor neuron phenotype involving the proximal segments of the upper limbs. The clinical course was slowly progressive, according to previous evidence of longer survival in patients with a lower motor neuron phenotype (12).

Genetic analysis showed a heterozygous p.D90A mutation of the *SOD1* gene.

To date, five Italian patients with the heterozygous p.D90A have been reported (6–9) (Table 1). They had a spinal onset involving lower and/or upper limbs typically with muscle weakness at an average age of 46, 6 years. Neurological examination showed both upper and lower motor neuron signs. Two patients complained of sensory disturbances. The disease onset did not show vocal cord paralysis in any patient except our case (6–9). Although this variant is not associated with a distinct phenotype, Origone et al. concluded that patients carrying the heterozygous p.D90A mutation have atypical clinical features. So, they suggested to perform molecular analysis looking for *SOD1* mutations in both SALS and FALS patients with atypical disease onset (8).

**Table 1:** Clinical features of ALS patients carrying the heterozygous p.D90A-*SOD1* mutation in the Italian population.

	**Sex**	**Familiarity**	**Age of onset (years)**	**Site of onset**	**Type of onset**	**Early sensory symptoms**	**Bulbar involvement**
Battistini et al. (6)	M	Yes	42	Spinal (lower limbs)	Sensory-motor neuropathy	Yes	No
Luigetti et al. (9)	M	No	45	Spinal (lower limbs)	Fasciculations Gait impairment	No	Pseudobulbar sign
Origone et al. (8)	M	No	41	Spinal (lower limbs)	Weakness Gait impairment	No	Spastic dysarthria Moderate dysphagia
Giannini et al. (7)	M	No	58	Spinal	Weakness and atrophy of the left hand Leg cramps	No	No
Giannini et al. (7)	F	No	47	Spinal	Foot drop	Yes	Dysarthria Dysphagia

*M, male; F, female*.

Although voice disturbances are rare, they are an insidious type of ALS onset (11).

Laryngological symptoms included: hoarseness, dysphonia, hypophonia/aphonia, non-productive cough, and life-threatening conditions as inspiratory stridor and laryngospasm.

Chen and Garrett demonstrated that a significant number of ALS patients with bulbar onset are initially referred to otolaryngologists. One thousand seven hundred fifty-nine patients were evaluated at a voice center from 1998 to 2003: <1% later received a diagnosis of ALS. In contrast, 20% of 220 ALS patients seen at the neurological clinic had bulbar onset and about half of them complained of dysphonia. When dysphonia was found, patients were initially referred to an otolaryngologist rather than a neurologist. Unfortunately, they were often misdiagnosed due to previous misleading diagnoses, dysarthria mistaken for dysphonia, subtle symptoms, and signs of neuromuscular disease overlooked by physicians. Therefore, the authors concluded that ALS diagnosis is a significative challenge in the otolaryngology practice (11).

Vocal cord problems may present with acute dyspnoea due to glottic narrowing or even glottic occlusion (10). In ALS, glottic narrowing has been supposed to depend on two types of vocal cord dysfunction: the supranuclear non-paralytic type causing an overactivity of vocal cord adductors and/or the infranuclear paralytic type consisting in neurogenic atrophy and weakness of the posterior cricoarytenoid muscle (10). Glottic narrowing is clinically symptomatic, resulting in inspiratory stridor, especially at an early disease stage in patients with a good vital capacity (10).

Laryngospasm is defined as a rapid and involuntary closure of the larynx (10). It is well-known that it may occur during intubation/extubation procedures and during disease progression probably due to a combination of gastro-esophageal reflux disease (GORD), aspiration of gastric contents, and dysphagia (10, 19). Diet modifications and pharmacological therapy with prokinetic agents and proton pump inhibitors (PPI) are recommended (19).

Instrumental assessment consists in laryngoscopy and laryngeal electromyography (10).

Changing to an upright position of the trunk, stabilizing the body through the fixation of the arms and slowly breathing are sufficient maneuvers to shorten the spasm episode (19).

Treatment of life-threatening vocal cord disturbances ranges from intubation and cricothyroidotomy to tracheotomy according to patient's advance directives (10).

Among neuromuscular diseases, laryngospasm is common at an early stage of Kennedy disease, while it is essentially a rare type of ALS onset (20).

In addition to our patient, literature reported other cases of ALS patients experiencing laryngospasm as initial manifestation of ALS (10, 11, 20).

It should be emphasized that vocal cord dysfunction occurs also in ALS patients without major bulbar involvement, as in the case of our patient (10, 21).

SALS and FALS patients presenting with laryngological onset has been reported to carry a missense mutation in the *SOD1* gene, as shown in Table 2 (13–18).

**Table 2:** Previously reported ALS patients carrying missense *SOD1* mutations and presenting with voice disturbance.

**Variant in protein/reference**	**p.I149T** **Fukae et al. (13)**	**p.D101Y** **Tan et al. (14)**	**p.A4V** **Salameh et al. (15)**	**p.I151T** **Kostrzewa et al. (16)**	**p.I113F** **Hermann et al. (17)**	**p.G147S** **Origone et al. (18)**
Familial/Sporadic ALS	FALS	FALS	FALS	FALS	FALS	SALS
Sex	F	M	M	F	M	M
Age at onset (yy)	43	57	73	48	49	56
Type of laryngological onset	Hoarseness due to bilateral vocal cord paralysis	Hoarseness due to bilateral vocal cord paralysis	Slurred voice	Dysarthria and dysphagia	Hoarseness due to bilateral vocal cord paralysis	Episodic dyspnoea and hoarseness due to bilateral vocal cord paralysis (right vocal cord at first)
Clinical features	Severe bulbar palsy, diffuse fasciculations, brisk deep tendon reflex, preserved limbs muscle strength	Dysphagia, progressive muscle weakness, and wasting in the right upper arm and on the right side of the face and tongue, atrophy of both scapular regions and the precordium, normal deep tendon reflexes	Right vocal cord paralysis, facial diplegia, dysarthria, dyspnoea, dysphagia, proximal right arm and mild proximal right leg weakness, arms fasciculations, normal/hyporeflexic deep tendon reflexes	Asymmetrical tetraparesis with muscle atrophy and fasciculations, brisk deep tendon reflexes	Muscle weakness in the proximal segments of the upper limbs, fasciculations of the tongue and shoulder muscles, brisk deep tendon reflexes	Progressive dyspnoea, inspiratory stridor, muscle weakness in four limbs, atrophy of small hand muscles, diffuse fasciculations, dysphagia, normal deep tendon reflexes
Progression	Tracheostomy 17 months after disease onset	Death 11 months after disease onset due to respiratory failure	Death 14 months after disease onset	Severe bulbar palsy and respiratory weakness	Death 15 months after disease onset	Death 8 months after disease onset due to respiratory failure

*FALS, Familiar Amyotrophic Lateral Sclerosis; SALS, Sporadic Amyotrophic Lateral Sclerosis; yy, years; M, male; F, female*.

Hermann et al. described a patient with hoarseness and muscle weakness in the proximal segments of the upper limbs at onset. As our patient, he had a bilateral compromission of vocal folds and a predominant lower motor neuron phenotype although upper motor neuron signs were present too. However, the disease progression was different from our case and the patient died 15 months after disease onset. Genetic analysis revealed a heterozygous missense mutation c.337 A>T in exon 4 of the *SOD1* gene. Authors suggested a pathogenic role for this mutation (17).

Table 2 summarizes other patients affected by motor neuron disease with voice disturbances as disease onset. Most of them presented with hoarseness and had a rapid progressive disease course and none of them carried the same mutation as our case. Some of them showed a predominant lower motor neuron phenotype (14, 15, 18).

## Conclusion

ALS onset may be insidious and subtle including laryngological symptoms like hoarseness, dysphonia, inspiratory stridor, and laryngospasm. Therefore, many ALS patients are initially referred to an otolaryngologist rather than a neurologist. Although the role of genetic testing is still controversial in clinical practice, our findings suggest that molecular analysis of the *SOD1* gene may be indicated in ALS patients with laryngological onset.

## Data Availability Statement

Some of the original contributions presented in the study are publicly available. This data can be found here: ZENODO: https://zenodo.org/record/5361094. The rest of the data can be provided by request to the corresponding author.

## Ethics Statement

Written informed consent was obtained from the individual for the publication of any potentially identifiable images or data included in this article.

## Author Contributions

GC, LD, EA, and MC contributed to writing of the manuscript and to critical revision of the manuscript. CC and IP contributed to genetic analysis. All authors gave important contributions to the final form of the manuscript.

## Funding

We want to thank the Italian Ministry of Health (Ricerca Corrente 2020-2021).

## Conflict of Interest

The authors declare that the research was conducted in the absence of any commercial or financial relationships that could be construed as a potential conflict of interest.

## Publisher's Note

All claims expressed in this article are solely those of the authors and do not necessarily represent those of their affiliated organizations, or those of the publisher, the editors and the reviewers. Any product that may be evaluated in this article, or claim that may be made by its manufacturer, is not guaranteed or endorsed by the publisher.
