# Supplementary material for: Case Report: Laryngospasm as Initial Manifestation of Amyotrophic Lateral Sclerosis in a Long-Survival Patient With Heterozygous p.D90A – SOD1 Mutation
Source: Front Neurol. 2021 Sep 30;12:708885. doi: 10.3389/fneur.2021.708885 (PMC8514733; doi:10.3389/fneur.2021.708885)
Supplement: Supplementary file 1 [file Data_Sheet_1.docx]

Supplementary Material

# Supplementary Data

# Genetic testing

# After obtaining a written informed consent, DNA was isolated from peripheral blood cells and quantified with NanoDrop ND1000 UV-Vis Spectrophotometer and Qubit® fluorometer (Thermo Scientific). Next Generation Sequencing (NGS) analysis (SureSelectQXT Target Enrichment for Illumina Multiplexed Sequencing, Agilent Technologies) was performed, using a customized panel of 174 genes related to neurodegenerative diseases. Genes inserted in the customized panel are: AARS, ABCA7, ABCD1, ACTA1, ADH1C, ADORA1, AGBL5, ALS2, AMBRA1, ANG, AP5Z1, APEX1, APP, AR, ARHGEF28, ASAH1, ATL1, ATP13A2, ATP1A3, ATP2B3, ATXN1, ATXN2, ATXN3, BAG3, BRWD3, BSCL2, C9ORF72, CCS, CHCHD10, CHCHD2, CHMP2B, CLN8, COQ2, CRYM, CUL4B, CXCL8, CYP27A1, CYP7B1, DAO, DCTN1, DHTKD1, DNAJC12, DNAJC6, DPP6, DTNBP1, DYNC1H1, EDN1, EIF4G1, ELOVL7, ELP3, EPHA4, ERBB4, EWSR1, FA2H, FANCL, FBXO47, FBXO7, FEZF2, FGGY, FIG4, FUS, GARS, GBA, GBE1, GCH1, GDAP1, GIGYF2, NAT8, GLE1, GPNMB, GRN, HEXA, HFE, HGSNAT, HNRNPA1, HNRNPA2B1, HNRNPA3, HSPB3, HSPB8, ICAM1, IGHMBP2, IL1A, IL1B, ITPR2, KIF1A, KIF5A, KIFAP3, L1CAM, LIF, LRRK2, METAP2, MAPT, MATR3, MFN2, MRS2, NAIP, NEFH, NEK1, NOTCH3, OPA1, OPA3, OPTN, PANK2, PARK2, PARK7, PDYN, PFN1, PINK1 PLA2G6, PLEKHB2, PLP1, PNPLA6, POLG, PON1, PON2, PON3, PRNP, PRPH, PSEN1, PSEN2, PTRHD1, RAB39B, REEP1, RNF19A, SARM1, SETX, SCH2, SIGMAR1, SLC1A2, SLC1A4, SLC2A1, SLC52A1, SLC52A2, SLC52A3, SMN1, SNCA, SOD1, SORL1, SPAST, SPG11, SPG21, SPG7, TACR1, SPTLC1, SQSTM1, SS18L1, SYNE1, SYNJ1, TAF1, TAF15, TARDBP, TBK1, TNF, TOMM40, TREM2, TRIM28, TRPV4, TUBA4A, TUBB4A, UBA1, UBE2A, UBE3A, UBQLN2, UNC13A, UCHL1, VAPB, VCP, VEGFA, VPS13A, VPS13C, VPS35, VPS54, ZFYVE26, ZHX2. FastQ files generation was performed using MiniSeq provided software (Real Time Analysis RTA v.1.18.54 and Casava v.1.8.2, Illumina, Inc., San Diego, CA). FastQ files provided for each sample, containing mate paired-end reads after demultiplexing, were trimmed for adapter removal with cutadapt (v1.10). Trimmed FastQ files were aligned to hg19 reference genome exploiting the Burrows-Wheeler transformation-based alignment via BWA-mem software v7.5a (1). BAM files were sorted and indexed via samtools v1.19 and Picard-tools v1.95 (http://broadinstitute.github.io/picard/). GATK V3.1 was used for insertions/deletions realignment (with RealignTargetCreator, IndelRealigner and BaseRecalibrator) and variant calling (with UnifiedGenotyper) according to GATK Best Practices recommendations (2-4). Produced VCF were processed with eVAI software (enGenome, Pavia, Italy) for annotation and variant classification. Through NGS analysis we identified the heterozygote variant g.12669A>C, c.272A>C in the exon 4 of the SOD1 gene resulting in the amino acid change p.Asp91Ala. The c.272A>C mutation was then confirmed by Sanger sequencing on ABI 3500 Genetic Analyzer (Applied Biosystems). The forward and reverse primers were 5′GAAGCCTTGTTTGAAGAGC3′ and 5′AACCGCGACTAACAATCAA3′ respectively (Metabion International AG, Germany) and PCR cycling conditions were 95 °C for 4 min, followed by 38 cycles of 95 °C for 30 s, 58 °C for 30 s, 72 °C for 30 s, with a final extension step of 7 min at 72 °C. Mutation nomenclature is based on RefSeq NM_000454.4 [GenBank] (considering the A of the ATG as nt 1) and on RefSeq NG_008689.1 for SOD1 cDNA and genomic sequences respectively, and follows the guidelines of the Human Genome Variation Society (http://www.hgvs.org/mutnomen/).

# Supplementary References

# 1. Li H, Durbin R. Fast and accurate short read alignment with Burrows-Wheeler transform. Bioinformatics. 2009 Jul;25(14):1754–60.

# 2. Li H, Handsaker B, Wysoker A, Fennell T, Ruan J, Homer N, et al. The Sequence Alignment/Map format and SAMtools. Bioinformatics. 2009 Aug;25(16):2078–9.

# 3. Van der Auwera GA, Carneiro MO, Hartl C, Poplin R, del Angel G, Levy-Moonshine A, et al. From fastQ data to high-confidence variant calls: The genome analysis toolkit best practices pipeline. Curr Protoc Bioinforma. 2013;(SUPL.43).

# 4. Depristo MA, Banks E, Poplin R, Garimella K V., Maguire JR, Hartl C, et al. A framework for variation discovery and genotyping using next-generation DNA sequencing data. Nat Genet. 2011 May;43(5):491–501.
